# Voxel-based analysis of ^201^Tl SPECT for grading and diagnostic accuracy of gliomas: comparison with ROI analysis

**DOI:** 10.1007/s12149-013-0711-y

**Published:** 2013-04-17

**Authors:** Tomoyuki Kuwako, Sunao Mizumura, Ryusuke Murakami, Tamiko Yoshida, Masato Shiiba, Hidetaka Sato, Yoshimitsu Fukushima, Akira Teramoto, Shin-ichiro Kumita

**Affiliations:** 1 Department of Radiology, Nippon Medical School, 1-1-5 Sendagi, Bunkyo-ku, Tokyo, 113-8603 Japan; 2 Department of Radiology, Toho University Omori Medical Center, 6-11-1 Omorinishi, Ota-ku, Tokyo, 143-8541 Japan; 3 Department of Diagnostic Imaging Center, Toranomon Hospital, 1-2-3 Toranomon, Minato-ku, Tokyo, 105-0001 Japan; 4 Department of Neurosurgery, Nippon Medical School, 1-1-5 Sendagi, Bunkyo-ku, Tokyo, 113-8603 Japan

**Keywords:** ^201^Tl SPECT, Glioma, Voxel-based analysis

## Abstract

**Purpose:**

The aim of this retrospective study was to assess the utility of a voxel-based analysis (VBA) method for ^201^Tl SPECT in glioma, compared to conventional ROI analysis.

**Methods:**

We recruited 24 patients with glioma (high-grade 15; low-grade 9), for whom pre-operative ^201^Tl SPECT and MRI were performed. SPECT images were coregistered with MRI. The uptake ratio (UR) images of tumor to contralateral normal tissue were measured on early and delayed images, and the ^201^Tl retention index (RI) map was calculated from the early and delayed uptake ratio maps. In the ROI analysis, tumors were traced on a UR map, and the mean and maximal uptake ratio values on the early images were, respectively, defined as the mean and maximal UR. The mean and maximal RI values (mean and maximal RI) were calculated by division of the mean and maximal UR, respectively, on the delayed image by the mean and maximal UR on the early image. For the RI map calculated voxel by voxel, the maximal RI value was defined as VBA-RI. We evaluated sensitivity and accuracy of differential analysis with the mean and maximal UR, RI, and VBA-RI.

**Results:**

The high- and low-grade groups showed no significant difference in mean and maximal RI (0.98 ± 0.12 vs. 1.05 ± 0.09 and 0.98 ± 0.18 vs. 1.05 ± 0.14, respectively). The AUC and accuracy of the mean and maximal RI were 0.681 and 66.7 %, and 0.622 and 62.5 %, respectively. In contrast, VBA-RI was higher in high-grade than in low-grade glioma (1.69 ± 0.27 vs. 0.68 ± 0.66, *p* < 0.001). The AUC and accuracy of VBA-RI were 0.963 and 95.8 %, which are higher than those obtained for mean (*p* < 0.05) and maximal RI (*p* < 0.01). There was no significant difference in ROC between the VBA-RI and the mean UR (0.911, *p* = 0.456) and maximal UR (0.933, *p* = 0.639); however, the AUC, sensitivity, and diagnostic accuracy of VBA-RI were all higher than those of the mean and maximal UR.

**Conclusion:**

The voxel-based analysis method of ^201^Tl SPECT may improve diagnostic performance for gliomas, compared with ROI analysis.

## Introduction

Thallium-201 chloride single photon emission computed tomography (^201^Tl SPECT) is widely available for brain tumors. This technique has been used for the grading of tumor malignancy [1–4] and differentiation of tumor recurrence from radiation necrosis [5–7]. ^201^Tl behaves like potassium, and relies on the sodium–potassium adenosine triphosphatase membrane transport system, as well as regional brain blood flow, reflecting the permeability of the blood–brain barrier, tissue viability, and cellular uptake [8–10]. For differential diagnosis and malignancy grading, however, although the important findings of ^201^Tl images have conventionally been considered to be high uptake on early phase ^201^Tl images and high retention indices (RI) for ^201^Tl uptake in the delayed phase relative to that in the early phase, the results have been controversial. Some authors have found that there is no statistically significant difference in ^201^Tl retention for histological malignancies [11, 12]. In contrast, other authors have shown the ^201^Tl RI to be correlated with the histological grading of brain tumors [3, 4, 6, 13]. These studies recruited tumors of differing histology, including gliomas as well as metastatic tumors, meningiomas, etc. Especially, meningiomas should be evaluated separately from gliomas, as extra-axial tumors may display different ^201^Tl kinetics from intra-axial tumors, due to their unique hemodynamics which proceeds without a blood–brain barrier [12].

For the semiquantitative analysis of ^201^Tl images with brain tumors, the region of interest (ROI) analysis has been widely discussed in previous reports; however, this method presents some problems. Firstly, ROI definition on tomographic images has poor reproducibility, especially for small lesions, due to the low resolution of ^201^Tl images. Secondly, in heterogeneous tumors (e.g., containing necrotic and cystic components) an ROI analysis may underestimate the activity of the tumor tissue, and misinterpret its grading [14, 15]. Finally, ROI analysis is not suitable for coregistration of two phase images (early and delayed images) for calculation of the retention indices, and cannot assess the ^201^Tl distribution in the whole brain.

On the other hand, a voxel-based analysis (VBA), which is essentially voxel-by-voxel comparison, may resolve these problems. In functional images (PET, SPECT), or structural images (3-dimensional MRI), this method is usually used for statistical analysis in the whole brain, without a priori assumptions [16–19]. This method has automated coregistration, and supports comparison with different images in the whole brain, on a voxel-by-voxel basis. Furthermore, even if the tumor uptake is heterogeneous, the voxel-based analysis method reveals the activities of the tumor tissue on a per-voxel basis, and evaluates its malignancy, selectively. The purpose of this study was to estimate the ^201^Tl retention index using a voxel-based analysis method for the grading and diagnostic accuracy of supratentorial gliomas, and to compare the results with those of the ROI analysis method.

## Materials and methods

### Patients

In this study, from October 2005 to March 2009, 24 consecutive patients (16 males and 8 females) with pre-operative supratentorial gliomas received ^201^Tl SPECT imaging and MR imaging at our institution (Table 1). All patients were given informed consent, and we obtained institutional approval for the retrospective analysis. Both techniques were performed within 1 week. Histological diagnosis was obtained by open surgery (*n* = 20) or biopsy (*n* = 4). Twenty-four tumors consisted of 10 glioblastomas, 5 anaplastic gliomas (2 anaplastic astrocytomas, 2 anaplastic oligoastrocytomas, and 1 anaplastic ependymoma), 2 diffuse astrocytomas, 2 oligodendrogliomas, 1 oligoastrocytoma, 1 pilocytic astrocytoma, and 3 astrocytomas which were consistent with Grade II astrocytoma; however, their specific histologies were unknown. To facilitate comparison, the various histological glioma entities were assigned to two groups: a high-grade group (*n* = 15) consisted of glioblastoma (Grade IV) and anaplastic glioma (Grade III), and a low-grade group (*n* = 9) consisted of diffuse astrocytoma (Grade II), oligoastrocytoma (Grade II), oligodendroglioma (Grade II), astrocytoma (Grade II), and pilocytic astrocytoma (Grade I).

**Table 1:** Characteristics of glioma patients

Pt	Sex	Age	Histology	Mean UR	Maximal UR	Mean RI	Maximal RI	VBA-RI
1	F	62	Glioblastoma IV	2.58	4.04	0.95	0.91	1.81
2	M	54	Glioblastoma IV	1.63	2.62	0.96	1.14	1.43
3	M	75	Glioblastoma IV	2.72	4.42	1.00	0.95	2.58
4	F	78	Glioblastoma IV	2.11	3.09	0.93	0.81	1.65
5	M	74	Glioblastoma IV	2.71	3.95	0.96	0.96	1.64
6	M	69	Glioblastoma IV	3.80	6.04	0.90	0.95	1.69
7	M	75	Glioblastoma IV	2.07	4.57	0.96	0.86	1.48
8	F	18	Glioblastoma IV	2.28	3.66	1.07	1.16	1.66
9	M	72	Glioblastoma IV	3.75	6.85	0.82	0.86	1.79
10	F	62	Glioblastoma IV	1.68	2.48	1.10	1.03	1.64
11	M	29	Anaplastic oligoastrocytoma III	1.85	2.80	1.13	1.21	1.78
12	M	55	Anaplastic oligoastrocytoma III	2.82	4.27	0.71	0.59	1.48
13	M	59	Anaplastic astrocytoma III	1.32	2.01	1.19	1.28	1.49
14	M	61	Anaplastic ependymoma III	2.74	5.51	0.98	0.88	1.57
15	M	65	Anaplastic astrocytoma III	1.40	2.42	1.07	1.15	1.68
16	M	66	Oligodendroglioma II	1.64	3.06	0.98	1.02	1.61
17	M	74	Astrocytoma II	1.42	1.82	1.12	1.04	1.25
18	M	13	Pilocytic astrocytoma I	1.95	2.55	0.86	0.88	0.98
19	F	71	Astrocytoma II	1.42	2.27	1.03	0.91	1.20
20	F	42	Oligoastrocytoma II	1.01	1.47	1.12	1.30	1.04
21	F	12	Oligodendroglioma II	0.91	1.21	1.04	1.05	0
22	M	61	Diffuse astrocytoma II	1.18	1.92	1.13	1.13	0
23	F	79	Astrocytoma II	0.86	1.34	1.05	1.23	0
24	M	56	Diffuse astrocytoma II	0.86	1.56	1.08	0.92	0

### Image acquisition

#### ^201^Tl SPECT

SPECT was performed using a triple-headed camera (PRISM 3000; Philips) equipped with a low-energy, fan beam collimator. Imaging was started from 10 min (early imaging) and from 4 h (delayed imaging) after intravenous injection of 111 MBq of ^201^Tl-chloride. We performed SPECT data collection (60 s/step, 60 steps) for 24 min. The matrix size was 128 × 128, and the collection window was 74 keV centered with 20 % width. Raw data were reconstructed with 2.2 × 2.2 × 6.7 mm^3^ in voxel size after decay correction using OS-EM (four iterations).

#### Magnetic resonance imaging

Magnetic resonance examination was performed using 1.5 or 3.0 T (Achieva NovaDual 1.5T and Achiva 3.0T; Philips Medical Systems, Best, the Netherlands; and Signa Cvi 1.5T; GE Medical Systems, Milwaukee, Wis.) with a standard head coil. The usual protocol includes T1-weighted, T2-weighted, FLAIR images and a T1-weighted image after intravenous injection of gadolinium-DTPA contrast (Gd-T1WI) to the following imaging parameters: slice thickness, 4.5 mm; gap, 2.5 mm; field of view (FOV), 240 × 240 mm; and, matrix size, 512 × 512.

#### Image processing of ^201^Tl SPECT (Fig. 1)

Firstly, early and delayed ^201^Tl image sets were coregistered with their corresponding Gd-T1WI images using customized BEAT-TL (Brain Easy Analysis Tool Thallium-201) software with image calculation (FUJIFILM RI Pharma Co., Ltd., Japan) [20] employing “statistical parametric mapping 2” (SPM2) (http://www.fil.ion.ucl.ac.uk/spm). ^201^Tl SPECT images were superimposed onto the Gd-T1WI images in 20 slices with a 256 × 256 matrix size, to inspect the agreement of tumor uptake with tumor location.

**Fig. 1:**
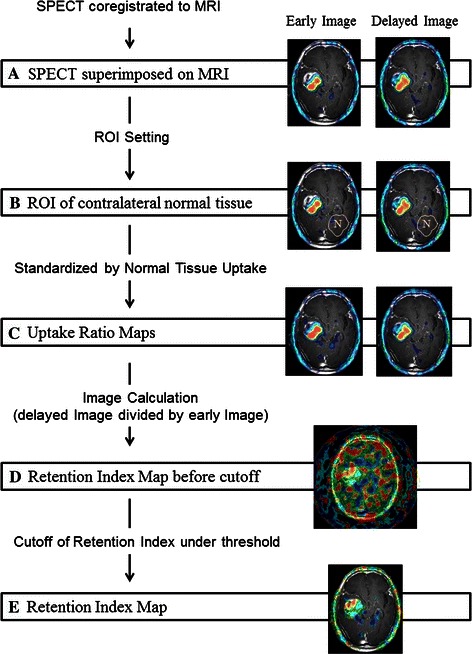
Image processing of ^201^Tl SPECT. **a** Early and delayed ^201^Tl SPECT, registered to and overlaid on the MRI image. The anatomical locations for the two phase images were matched on a per-voxel basis. **b** A normal brain ROI definition on the contralateral hemisphere. **c** Early and delayed ^201^Tl SPECT were standardized by the mean value for a contralateral normal tissue ROI on a per-voxel basis, resulting in an early and delayed uptake index map. **d** A retention index map before cutoff was calculated from the delayed index map divided by the early index map on a per-voxel basis. **e** The retention index map was completed by the replacement of voxel values under 1.5 with zero

Secondly, we selected a slice corresponding to the location of the brain tumor on MRI (Fig. 1a) [1]. Next, a normal brain ROI was drawn on the contralateral normal brain tissue in early and delayed images, with the same voxel size as each tumor ROI (Fig. 1b). Consequently, the coregistered early and delayed SPECT images were divided by the mean value of the contralateral normal tissue ROI, on a per-voxel basis in the whole brain. The obtained images were the early and delayed “uptake ratio maps” (Fig. 1c).

**Fig. 2:**
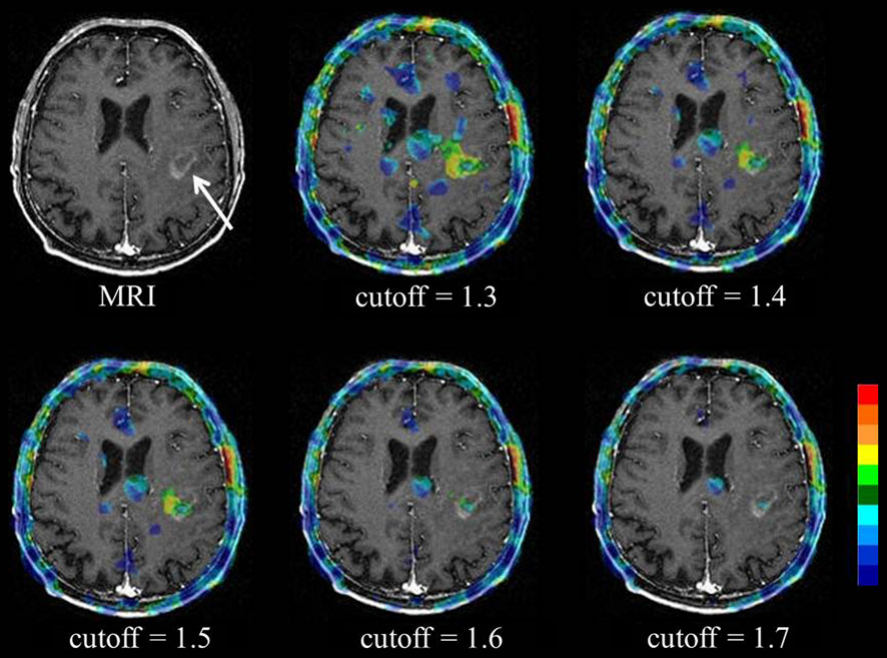
Cutoff value setting for retention index map. Low-grade (Grade II) astrocytoma on MR imaging (*arrow*). The background uptake and/or noise was widely remained for cutoff values of either 1.3 and 1.4. On the other hand, a part of the tumor uptake was removed for cutoff values of either 1.6 or 1.7

Finally, for the retention index map, the delayed uptake ratio map was divided by the early uptake ratio map, on a per-voxel basis (Fig. 1d). To remove the background uptake and/or noise, uptake values lower than 1.5 among whole brain voxels on the early and/or delayed uptake ratio maps were then replaced with zero (Fig. 1e). This cutoff value was determined by visual assessment in all cases (mean ± SD 1.50 ± 0.06; range 1.4–1.7), so as to keep the tumor uptake as well as remove the background uptake and/or noise (Fig. 2).

#### ROI analysis of ^201^Tl SPECT

ROI analysis was performed according to previous reports [1]. The coregistered early and delayed ^201^Tl images were used (Fig. 1a). The ROI for the brain tumor was manually placed over the areas of abnormal ^201^Tl uptake determined according to the MRI findings. As mentioned above, a normal brain ROI was drawn on the contralateral brain tissue with the same voxel size as for the corresponding tumor ROI.

The ratio of the mean tumor count to normal brain count, and that of the peak tumor count to normal count were calculated as the mean and maximal uptake ratios (mean and maximal URs), respectively. The ratios of the mean and maximal URs on the delayed image to those on the early image were calculated as the mean and maximal retention indexes (mean and maximal RIs). Briefly, the four parameters in the ROI analysis were defined, as follows:

1. mean UR = (mean count in tumor ROI/mean count in normal tissue) on the early image;
2. maximal UR = (peak count in tumor ROI/mean count in normal tissue) on the early image;
3. mean RI = mean UR on the delayed image/mean UR on the early image;
4. maximal RI = maximal UR on the delayed image/maximal UR on the early image.

Note that the voxel with the peak count in the tumor ROI on early image does not necessarily correspond to that on delayed image.

#### Voxel-based analysis of ^201^Tl SPECT

Voxel-based analysis was performed using previously obtained “the retention index map”. The retention index map was created as follows: firstly, early and delayed ^201^Tl SPECT images were registered on their corresponding MR images. Thus, all the voxels between early and delayed ^201^Tl SPECT images had the same anatomical locations in the whole brain. Secondly, “uptake ratio maps” were calculated by normalizing to mean uptake in the contralateral ROI. Thirdly, “retention index map before cutoff” was created from two uptake ratio maps (delayed uptake ratio map divide by early uptake ratio map on a per-voxel basis). Finally, “retention index map” was completed by the replacement of voxel value under 1.5 with zero for the elimination of the background uptake and/or noise.

The resulting parameter, the “VBA-RI” was defined as the peak value for a tumor in the “retention index map” obtained after the image processing. Subsequently, we confirmed that the majority of VBA-RI locations corresponded to tumors, with the exception of 5 cases. In 3 of these cases, tumor accumulations were not clear (Pt 20–24), while in the other cases, tumors existed near the scalp (Pt 12, 18). For this reason, these 5 cases required interpretation as a brain tumor using coregistered MRI in a complementary manner.

### Statistical analysis

All of the UR and RI parameters were presented as mean ± standard deviation and statistically compared to the high- and low-grade groups with the Mann–Whitney *U* test. Receiver operating characteristic curves were employed to differentiate the high and low-grade glioma groups, and identify the cutoff values of the five indices. A *p* value <0.05 was considered significant.

## Results

### ROI analysis

**Fig. 3:**
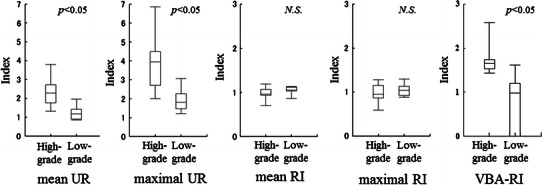
Box plot of each of the indices in the high-grade group vs. the low-grade group. The mean and maximal UR shows a significant difference between the high- and low-grade groups. Although the VBA-RI also differed for the two groups, the mean and maximal RIs did not

**Fig. 4:**
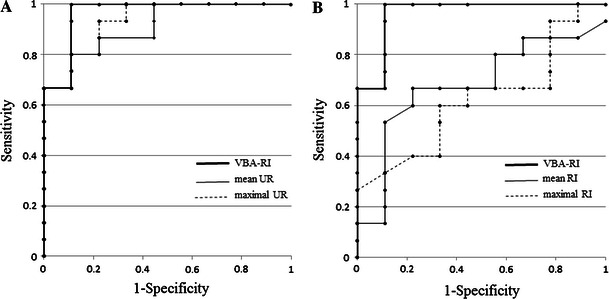
ROC analysis of VBA-RI and mean and maximal URs. **a** Receiver operating characteristic curves (ROC) for VBA-RI and mean and maximal URs. **b** ROCs for VBA-RI and mean and maximal RIs. The AUC for the mean and maximal URs, and VBA-RI were 0.911, 0.933, and 0.970, respectively, which were higher than those of mean and maximal RIs (0.681 and 0.622). The AUC and accuracy of VBA-RI were significantly higher than those of the mean RI (*p* < 0.05) and maximal RI (*p* < 0.01). The AUC of VBA-RI did not significantly differ from those of the mean and maximal URs. However, the AUC, sensitivity, specificity, and diagnostic accuracy were all higher for VBA-RI, relative to both the mean UR and maximal UR

The mean and maximal UR values were significantly higher in high-grade group, relative to the low-grade group (mean UR: 2.36 ± 0.76 vs. 1.25 ± 0.38, *p* < 0.001; maximal UR: 3.92 ± 1.42 vs. 1.91 ± 0.61, *p* < 0.001) (Fig. 3). In the ROC analysis, mean and maximal URs had cutoff values of 1.42 and 2.27, with AUC values of 0.911 and 0.933, respectively (Fig. 4a). The sensitivity and accuracy were 86.7 and 83.3 % for the mean UR, and 93.3 and 87.5 % for the maximal UR.

There were no significant differences in mean and maximal RI between high- and low-grade groups (mean RI: 0.98 ± 0.12 vs. 1.05 ± 0.09, *p* = 0.168; maximal RI: 0.98 ± 0.18 vs. 1.05 ± 0.14, *p* = 0.322) (Fig. 3). The AUC values for the mean and maximal RI were 0.681 and 0.622, respectively (Fig. 4b). The sensitivity and accuracy were 66.7 and 66.7 % for mean RI, and 66.7 and 62.5 % for maximal RI.

### Voxel-based analysis

The VBA-RI was significantly higher in the high-grade group, relative to the low-grade group (1.69 ± 0.27 vs. 0.68 ± 0.66; *p* < 0.001) (Fig. 3). In the ROC analysis, the VBA-RI had a cutoff value of 1.25 and an AUC of 0.963 (Fig. 4a, b), with a sensitivity and accuracy of 100 and 95.8 %, respectively.

**Fig. 5:**
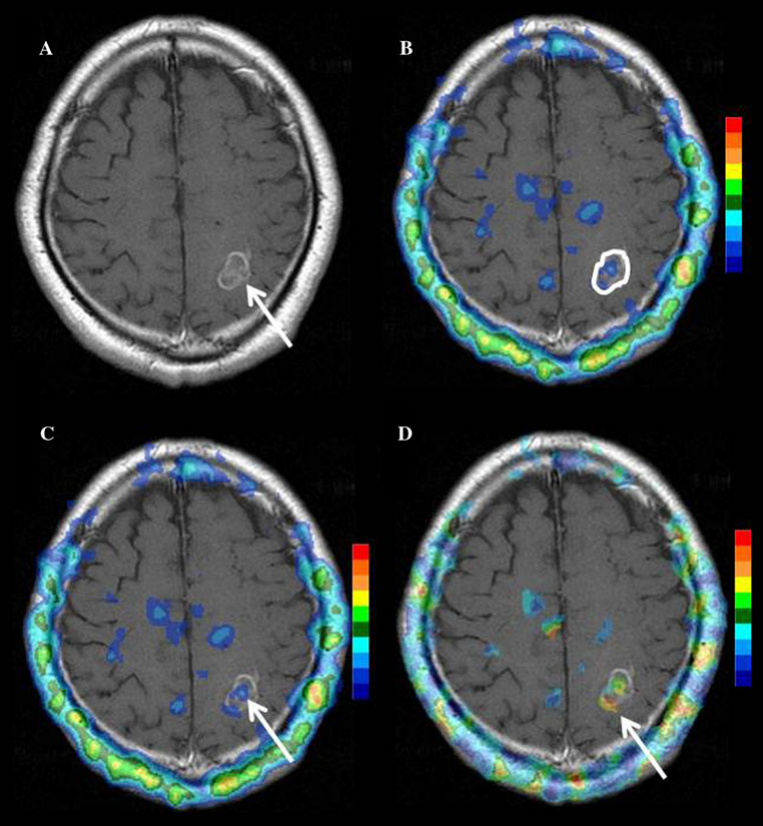
Case 1 is a 55-year-old male patient who presented with a high-grade (grade III) tumor in the left parietal oligoastrocytoma. **a** Gadolinium-DTPA enhanced T1 weighted image; **b** mean UR image; **c** maximal UR image; **d** VBA-RI image. The mean and maximal URs of the tumor demonstrated low values of 1.32 and 2.01, respectively, which were suggestive of low-grade glioma. The VBA-RI of the tumor, however, showed a high value of 1.49, which was suggestive of, and adequately in agreement with high-grade glioma

**Fig. 6:**
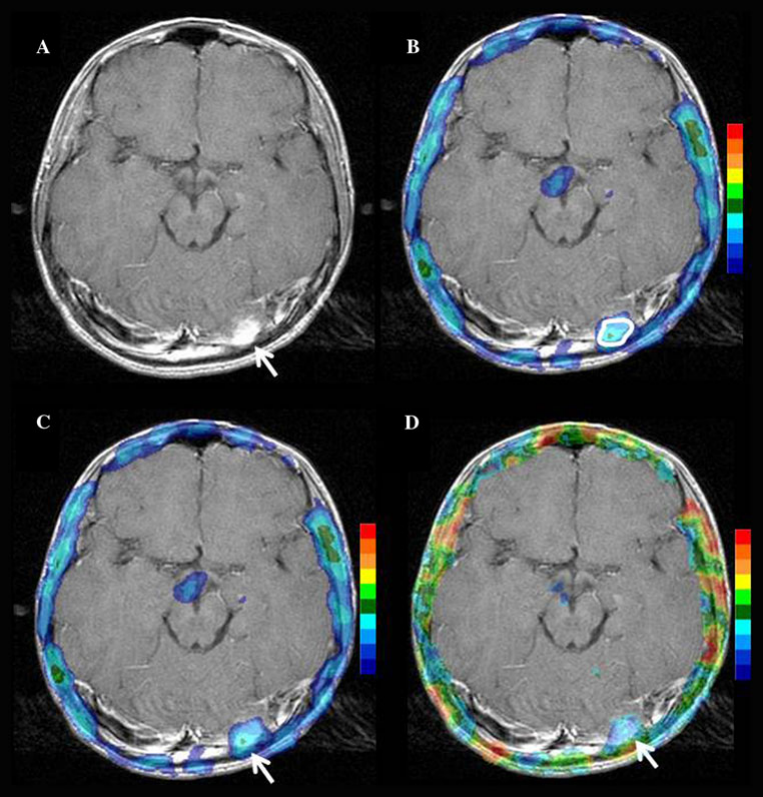
Case 2 is a 13-year-old male patient who presented with a low-grade (grade I) in left occipital pilocytic astrocytoma. **a** Gadolinium-DTPA enhanced T1 weighted image; **b** mean UR image; **c** maximal UR image; **d** VBA-RI image. Since the tumor existed near the scalp, the MRI was used for reference. The mean and maximal URs of the tumor demonstrated high values of 1.95 and 2.55, respectively, which were suggestive of high-grade glioma. The VBA-RI of the tumor, however, showed a low value of 0.98, which was suggestive of, and in agreement with low-grade glioma

Both the AUC and accuracy of VBA-RI were higher than those obtained for mean RI (*p* < 0.05) and maximal RI (*p* < 0.01). There was no significant difference in the AUC between VBA-RI and mean UR (*p* = 0.456), or between VBA-RI and maximal UR (*p* = 0.639). However, VBA-RI displayed higher AUC, sensitivity, and accuracy than either mean or maximal UR. Two representative patients are shown in Fig. 5 (Pt 13) and Fig. 6 (Pt 18).

## Discussion

^201^Tl uptake and its retention index (which reflects ^201^Tl affinity) have often been evaluated for the diagnosis of brain tumors using the ROI method. However, the diagnostic criteria of these parameters for the diagnosis of brain tumors have not been established adequately using the ROI method. Some reports have mainly assessed the histological grading of glioma using only the ^201^Tl uptake ratio in early imaging using ROI analysis [9, 21–25]. Our results also demonstrated that the maximal and mean UR, using ROI analysis, show high accuracy (mean UR 83.3 %; maximal UR 87.5 %). The diagnostic accuracy, sensitivity, and AUC obtained with VBA-RI were superior to those obtained using the other parameters, although there were no significant differences among the accuracies of VBA-RI (95.8 %) and the mean UR and maximal UR. Therefore, the use of VBA-RI yielded improved diagnostic performance for gliomas.

Furthermore, a voxel-based analysis presents several additional advantages, in that does not require the definition of a tumor margin, and reveals voxel-by-voxel activity for inhomogeneous tumors. In this study, we attempted to calculate the retention index on a voxel-by-voxel basis using anatomical coregistration and the voxel-based analysis techniques which a statistical image analysis employs. We assumed that the maximal retention index reflects the ^201^Tl affinity of a brain tumor, which is related to tumor malignancy, even when the tumor has inhomogeneous components.

Interestingly, in our study, the voxel of maximal uptake in a brain tumor on an early image did not correspond to that on the delayed image in all cases. Thus, the RI obtained using the ROI method does not reflect the exact change in ^201^Tl uptake, in particular, for a tumor with an inhomogeneous distribution. Prieto et al. [26] reported the successful utilization of the voxel-based analysis of dual-time-point ^18^F-FDG PET images for brain tumors. They also considered that the location of the voxel of maximal uptake in an early image did not match the voxel of maximal uptake in the corresponding delayed image. A similar result was also obtained in our study using ^201^Tl SPECT. For maximal UR, we pointed out that the variation was computed between two different voxels in the tumor, which does not represent the real change in the voxel of maximal uptake in the early image. Therefore, the voxel-based retention index would be more meaningful for the characteristic ^201^Tl behavior in a brain tumor. In our results, the maximal RI underestimates the differences in maximal uptake between the early and delayed images, compared with the change estimated by RI using VBA analysis, and the diagnostic accuracy of maximal RI was inferior to that of VBA-RI (62.5 vs. 95.8 %). VBA-RI was significantly higher in the high-grade group than in the low-grade group, and displayed a very high accuracy for classification of histological grading, while the mean and maximal RI with an ROI analysis showed no significant differences between the high- and low-grade groups.

There are some limitations in this study. Firstly, we ignored the voxels with a lower uptake ratio in the early and delayed images for the elimination of background uptake and/or noise. It might also exclude a portion of the brain tumor. While the assessment of uptake ratios without a cutoff process was reported using ^18^F-FDG brain tumor PET [26], it was necessary to evaluate ^201^Tl SPECT via processing, because ^201^Tl SPECT displays more nonspecific accumulation than ^18^F-FDG PET. Secondly, there may be a minor error in the coregistration of ^201^Tl SPECT to non 3-dimensional MRI using SPM. In this study, we found no errors in any of the acquired fusion images of 3-dimensional ^201^Tl SPECT and MRI. Finally, all of the recruited cases had brain tumors in our study. Therefore, the present study included no true negative case, which could possibly have an effect on the diagnostic accuracy.

## Conclusion

We have evaluated the affinity of ^201^Tl for brain tumors by its retention and uptake using a voxel-based and ROI-based analysis. The voxel-based retention index demonstrated a significant improvement in the differential analysis of tumor malignancy, compared to ROI-based uptake and retention indices. Although this preliminary study should encourage the use of ^201^Tl SPECT for brain tumors, further study is required with a larger prospective series of patients.
